# The Emerging Role of Explainable Artificial Intelligence in EEG-Based Autism Research: A Systematic Review

**DOI:** 10.3390/neurosci7020041

**Published:** 2026-04-01

**Authors:** Maria Eugenia Martelli, Simone Colella, Roberta Meloni, Federica Gigliotti, Antonello Rosato, Massimo Panella, Carla Sogos

**Affiliations:** 1 Department of Human Neuroscience, Sapienza University of Rome, 00185 Rome, Italy; roberta.meloni@uniroma1.it (R.M.); federica.gig@uniroma1.it (F.G.); carla.sogos@uniroma1.it (C.S.); 2 Department of Information Engineering, Electronics and Telecommunications, Sapienza University of Rome, 00184 Rome, Italy; simone.colella@uniroma1.it (S.C.); antonello.rosato@uniroma1.it (A.R.); massimo.panella@uniroma1.it (M.P.)

**Keywords:** Autism Spectrum Disorder, electroencephalography, explainable Artificial Intelligence, EEG biomarkers, Deep Learning, interpretability

## Abstract

The increasing prevalence of Autism Spectrum Disorder (ASD) has intensified research efforts aimed at clarifying its neurobiological underpinnings. Electroencephalography (EEG) has enabled the identification of functional alterations in neuronal networks, contributing to the characterization of ASD-related brain dynamics and supporting the investigation of links between neural processes and behavioral impairments. In recent years, Artificial Intelligence (AI) methods have been increasingly applied to EEG analysis, allowing the extraction of complex, high-dimensional features. However, the limited interpretability of many AI-based models represents a major barrier to their clinical translation. To address this issue, Explainable Artificial Intelligence (XAI) approaches have emerged as promising tools to enhance model transparency and neurobiological interpretability. This systematic review examined studies explicitly applying XAI techniques to EEG or event-related potential data from individuals with ASD. A comprehensive literature search was conducted across multiple electronic databases up to November 2025. Studies were included if they involved ASD populations, electrophysiological data, and AI-based analytical approaches with explicit explainability components. Due to substantial methodological heterogeneity, a qualitative narrative synthesis was performed. Eleven studies met the inclusion criteria. Overall, included articles highlighted partially overlapping electrophysiological patterns involving spectral alterations, functional connectivity, and network organization; however, some studies also revealed marked heterogeneity in study design and limited clinical characterization. Consequently, they should be interpreted with caution, as the field remains at a preliminary stage. This review outlines current trends, methodological limitations, and key gaps in XAI-driven EEG research in ASD, and discusses future directions toward clinically meaningful and interpretable neurophysiological biomarkers. The review protocol was registered in PROSPERO (CRD420251231630).

## 1. Introduction

Autism Spectrum Disorder (ASD) is a neurodevelopmental condition characterized by impairments in several domains of social functioning, particularly social interaction and communication, as well as restricted and repetitive behaviors [1]. Over the past decades, the diagnosis of ASD has steadily increased, with current global prevalence estimates ranging between 0.6% and 1.0%, varying according to geographical area, socioeconomic context, and availability of healthcare services [2,3]. The clinical presentation of ASD is markedly heterogeneous, encompassing a wide range of symptoms that differ substantially in severity and configuration, resulting in distinct clinical profiles. Despite this variability, symptoms typically emerge in early childhood and significantly compromise multiple functional domains and overall quality of life [4,5]. Although the clinical manifestations may evolve across development, ASD is a lifelong condition; its persistence and phenotypic heterogeneity make early diagnosis both essential and particularly challenging. Furthermore, ASD affects individuals across all ethnicities and socioeconomic backgrounds, and its etiology is multifactorial, involving genetic, environmental, and neurobiological factors that remain only partially understood [6,7].

Current international diagnostic guidelines rely primarily on clinical and behavioral assessments based on semi-structured interviews and direct observation, such as the Autism Diagnostic Interview-Revised (ADI-R) [8] and the Autism Diagnostic Observation Schedule-Second Edition (ADOS-2) [9]. While these instruments represent the gold standard for ASD diagnosis, their sensitivity may be influenced by several factors, including clinician expertise, age at symptom manifestation, and individual cognitive and language abilities [10,11]. Moreover, reliance on the emergence of clearly observable behavioral symptoms often limits diagnostic accuracy during the earliest developmental stages, when clinical interventions are known to be most effective [12,13,14]. Consequently, there is growing interest within both clinical and neuroscientific communities in identifying objective biomarkers that can support diagnostic procedures and capture shared neurobiological substrates underlying the broad spectrum of ASD phenotypes [15,16].

Within this framework, electroencephalography (EEG) has emerged as a promising and widely accessible tool for the identification of objective neurophysiological biomarkers of ASD and atypical brain functioning. EEG is commonly used in the assessment of neurodevelopmental disorders, which often co-occur with neurological atypies, and is typically available in clinical settings. Moreover, EEG is a low-cost and non-invasive technique, making it particularly suitable for pediatric populations [17,18,19]. Owing to its high temporal resolution and relative ease of acquisition, EEG represents a valuable tool for investigating functional neural characteristics associated with ASD, facilitating the discrimination between autistic and neurotypical individuals and supporting diagnostic procedures [20,21]. A growing body of literature has documented atypical patterns of brain oscillatory activity, inter-regional functional connectivity, and event-related potentials (ERP) in individuals with ASD compared to typically developing (TD) peers [13,21]. In particular, several studies have reported an atypical spectral profile in ASD, commonly referred to as a “U-shaped” power distribution [22,23]. This pattern is characterized by increased power in low- and high-frequency bands, including delta (1–3 Hz), theta (4–7 Hz), beta (13–35 Hz), and gamma (>35 Hz), alongside a reduction in alpha power (8–12 Hz) [23,24,25,26]. These alterations have been associated with atypical neural development and are thought to contribute to the cognitive, social, and sensory impairments commonly observed in ASD [25,26,27,28,29,30]. Importantly, evidence from pediatric populations indicates that early alterations in functional connectivity, observable as early as three months of age, may predict later severity of autistic symptoms [22,31]. This finding underscores the potential role of EEG-derived measures as screening tools and as support for early diagnosis [17,32].

The increasing relevance of EEG in ASD research has driven the adoption of Artificial Intelligence (AI) methodologies, particularly Machine Learning (ML) and Deep Learning (DL) approaches, for the analysis of EEG recordings. These models are capable of processing large-scale, high-dimensional datasets and extracting complex patterns that may not be detectable through traditional analytical methods. By overcoming some of the limitations of conventional clinical and diagnostic tools [33,34,35], AI-based approaches have enabled the identification of latent neural signatures that differentiate individuals with ASD from neurotypical controls, achieving classification accuracies ranging from 80% to 98% [33,36]. Despite these promising findings, many AI models are affected by the so-called “black box” problem, as they offer limited insight into the mechanisms underlying their decision-making processes. In clinical settings, however, AI-based models must be interpretable, reproducible, and grounded in neurobiological plausibility in order to be considered reliable and clinically meaningful.

Explainable Artificial Intelligence (XAI) has been proposed as a strategy to address this limitation by enhancing model transparency and enabling the interpretation of algorithmic decision-making processes. In this manuscript, we distinguish between interpretability, explainability, and the broader concept of XAI. Interpretability refers to the intrinsic transparency of a model; that is, the extent to which its internal structure and parameters can be directly understood by human observers without additional processing. Explainability refers to the set of techniques designed to provide insight into the behavior of complex models, either by analyzing their internal representations or by approximating their decision logic. The term XAI is used here as an umbrella concept encompassing both inherently interpretable models and post hoc explanation methods. From a methodological perspective, XAI approaches can be broadly categorized into (i) intrinsically interpretable models, where transparency is embedded in the model design, and (ii) post hoc explanation methods applied after model training. Post hoc methods can further be divided into model-specific techniques, which require access to internal model parameters (e.g., gradient-based attribution), and model-agnostic techniques, which treat the model as a black box and rely solely on input–output behavior. Inherently interpretable models are necessarily model-specific, as their transparency depends on their internal structure [37,38,39,40].

In the context of EEG analysis, XAI approaches can offer valuable insights into which neural signal features, such as frequency bands, channels, or cortical regions, contribute most strongly to model predictions. Routine clinical EEG is primarily oriented toward the identification of overt pathological abnormalities and it is not designed to detect subtle electrophysiological patterns, reflecting atypical, yet non-pathological, neural functioning. In contrast, XAI-based methods enable comparisons of such patterns and support predictive modeling in population with neurodevelopmental disorders [34,41]. Recent studies have demonstrated the potential of XAI methods for clinical and diagnostic applications. In particular, inherently interpretable Convolutional Neural Networks (CNNs) and post hoc explanation techniques such as SHapley Additive exPlanations (SHAP) applied to EEG data have been employed to quantify the contribution of specific EEG features to ASD discrimination [21,33,41]. Notably, findings derived from these approaches are largely consistent with existing behavioral and neurophysiological evidence. Studies leveraging SHAP analyses have highlighted the role of atypical theta and alpha activity in individuals with ASD [33,41], thereby supporting the neurobiological relevance of the extracted features.

By leveraging objective neurophysiological data and increasing the transparency of AI-driven decision-making processes, XAI approaches hold considerable potential for improving both the timing and accuracy of ASD diagnosis, while also providing novel insights into neurophysiological subprofiles within the autism spectrum. Given the increasing emphasis on multidimensional frameworks that integrate subjective clinical assessments with objective biomarkers, such as genetic information and EEG measures, XAI represents a particularly promising avenue for supporting clinical decision-making [37,38,42]. Nevertheless, despite the growing body of research in this area, a comprehensive synthesis of studies applying XAI techniques to EEG data in ASD is still lacking. Existing investigations show substantial heterogeneity in sample characteristics, EEG features (e.g., frequency bands, power spectra, and ERPs), methodological designs, and applied XAI approaches.

This systematic review aims to address this gap by examining and synthesizing studies that apply XAI approaches to EEG data analysis in individuals with ASD. Specifically, the main contributions of this review are: (i) to provide a systematic overview of AI-based approaches applied to EEG data in ASD; (ii) to categorize these approaches by distinguishing between intrinsically interpretable models and black-box models augmented with post hoc explanation methods; (iii) to critically analyze current XAI techniques applied to EEG in ASD; and (iv) to discuss methodological gaps and future directions for interpretable and clinically grounded AI models.

## 2. Methods

### 2.1. Study Design and Reporting

**Figure 1:**
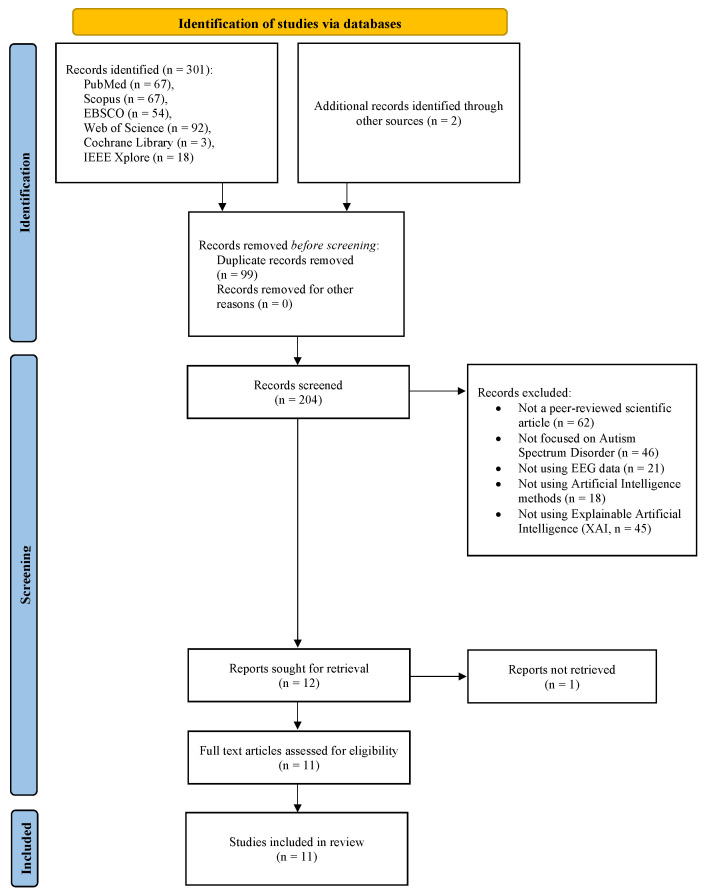
PRISMA flow diagram of the study selection process.

This review followed internationally recognized standards for the reporting of systematic reviews, as outlined in the Preferred Reporting Items for Systematic Reviews and Meta-Analyses (PRISMA) 2020 statement [43]. The study selection process is illustrated using a PRISMA flow diagram (Figure 1), detailing the identification, screening, eligibility, and inclusion of records. The PRISMA 2020 checklists are provided in the Supplementary Materials (Tables S1 and S2). Details of the protocol for this systematic review were registered in PROSPERO (registration number: CRD420251231630).

### 2.2. Eligibility Criteria

Studies were included if they met the following criteria: (a) original empirical research published in English or with an available English translation; (b) investigations conducted in populations diagnosed with ASD; (c) use of EEG data; and (d) application of AI-based methods, such as ML or DL approaches, incorporating explicit elements of model explainability or interpretability (e.g., XAI). Studies were excluded if they met any of the following criteria: (a) absence of participants with ASD; (b) lack of EEG data; (c) no explicit adoption of XAI methodologies; or (d) publication types including reviews, meta-analyses, books or book chapters, editorials, letters, commentaries, case reports, and case series.

To ensure conceptual precision, eligibility criteria were restricted to studies that explicitly adopted and described their interpretability strategies within a formal XAI framework. The aim of this review is not to survey all forms of model interpretability in EEG-based ASD research, but specifically to examine how XAI methodologies are implemented and evaluated in this domain. Approaches limited to feature importance reporting, ablation analyses, or other post hoc heuristic interpretations were not considered sufficient for inclusion unless they were explicitly embedded within a declared XAI framework. This distinction reflects the methodological scope of the review: XAI methods are characterized by structured explanatory mechanisms that aim to make model decision processes transparent, rather than by generic measures of variable relevance or model sensitivity.

### 2.3. Information Sources

A comprehensive literature search was conducted across the following databases to identify studies available up to November 2025: PubMed, Scopus, APA PsycArticles, APA PsycInfo, MEDLINE, CINAHL Plus with Full Text, Psychology and Behavioral Sciences Collection, Web of Science, Cochrane Library, and IEEE Xplore. In addition, reference lists of relevant reviews and included articles were manually screened to identify further eligible studies not captured through database searches. The final search across all sources was completed on 16 November 2025.

### 2.4. Search Strategy

A structured search strategy was implemented across all databases using combinations of terms related to electrophysiological measures (EEG/ERP), ASD, AI-based approaches, and XAI. Search terms included free-text keywords and database-specific fields, and were combined using Boolean operators (AND/OR). The strategy was adapted to the syntax and indexing requirements of each database (e.g., Title/Abstract, Keywords, All Fields). Keywords and controlled vocabulary terms (e.g., MeSH, where applicable) were included to maximize sensitivity. The complete search strings for each database are reported in Table 1.

### 2.5. Selection Process

Three reviewers independently assessed all retrieved records using a two-step selection process. Initially, titles and abstracts were evaluated for relevance; subsequently, potentially eligible articles underwent full-text assessment according to predefined inclusion and exclusion criteria. Exclusions at the full-text stage were recorded with explicit justification. Any disagreements were resolved through collegial discussion, with involvement of an additional reviewer when consensus was not immediately achieved. Overall, 301 records were identified. After screening and full-text assessment, 11 studies met the eligibility criteria and were included in the final synthesis.

### 2.6. Data Collection Process and Data Items

Data were independently extracted by three reviewers using a standardized Excel spreadsheet developed according to the PICO framework (Population, Intervention/Exposure, Comparison, Outcome). A fourth reviewer verified the extracted data for completeness and accuracy. The following information was collected for each included study: study design and methodological approach; country and research setting; sample characteristics (sample size, age range or mean age, sex distribution, and presence and type of control groups); ASD diagnostic criteria or assessment instruments; EEG acquisition characteristics (recording condition, task-based or resting-state paradigm, and number of electrodes); AI-based analytical methods (including ML or DL models); the type of XAI strategy adopted, distinguishing between intrinsically interpretable models and post hoc explanation techniques; main electrophysiological features analyzed (spectral, temporal, and functional connectivity measures); primary outcomes; and key findings. In addition, information regarding the level of clinical characterization (e.g., cognitive functioning, symptom severity, comorbidities, and medication status, when available) and reporting of socioeconomic and demographic variables was extracted. Additional bibliographic information included first author, year of publication, and country of origin.

### 2.7. Quality Assessment

Given the substantial heterogeneity of study designs and analytical approaches, a structured assessment of methodological rigor and potential sources of bias was performed by systematically evaluating multiple dimensions, including (i) diagnostic procedures, (ii) EEG preprocessing, (iii) validation frameworks, (iv) handling of class imbalance and confounding variables, as well as (v) the integration of XAI methods and (vi) code or data availability. All domains were assessed qualitatively and they were not weighted or prioritized. The criteria were applied systematically to all included studies and the results of this assessment are summarized in Table 2. This table enables comparison of methodological strengths, limitations, and potential sources of bias across the included studies.

### 2.8. Synthesis Methods

Given the substantial heterogeneity across study designs, participant characteristics, EEG paradigms, analytical approaches, and outcome measures, a quantitative meta-analysis was not feasible. Therefore, a qualitative narrative synthesis was conducted. Studies were grouped according to key methodological and conceptual dimensions relevant to the aims of the review, including: (1) EEG acquisition paradigm (resting-state versus task-based); (2) electrophysiological features analyzed (spectral, temporal, and functional connectivity measures); (3) AI methodologies (ML versus DL models); and (4) explainability or interpretability approaches applied. Within each domain, patterns of findings were summarized and compared across studies, with particular attention to convergent electrophysiological markers, consistency of model interpretability, and clinical relevance of the extracted features. This approach allowed for the integration of results across heterogeneous methodologies while highlighting methodological trends, sources of variability, and gaps in the existing literature.

## 3. Methodological Background and Analytical Framework

### 3.1. AI-Based EEG Studies in ASD

Before addressing XAI-oriented approaches, it is necessary to contextualize the evolution of AI-based methods applied to EEG analysis in ASD. A substantial body of literature has explored computational and learning-based techniques for modeling EEG signals in ASD without the explicit integration of formal XAI frameworks. Although these studies do not constitute the primary focus of the present systematic review, they provide essential methodological background and delineate the trajectory that led to the emergence of XAI-driven paradigms. Overall, this literature encompasses a broad spectrum of AI methodologies, ranging from traditional ML pipelines based on handcrafted EEG features to shallow NN models, DL architectures, and structured signal representations. Across these approaches, the predominant analytical task is binary classification between individuals with ASD and neurotypical controls, while a smaller subset of studies addresses regression-based formulations, symptom severity estimation, and task-specific cognitive or emotion recognition.

#### 3.1.1. Traditional Machine Learning Methods

Traditional ML represents the earliest and most widely adopted class of AI methods in EEG-based ASD research. These approaches typically rely on a two-stage pipeline, consisting of handcrafted feature extraction from EEG signals followed by supervised learning using conventional algorithms such as support vector machines (SVMs), random forests (RFs), k-nearest neighbors (k-NNs), or regularized linear models. Most traditional ML studies focused on spectral and statistical EEG descriptors, including band-limited power, power ratios, and other amplitude-based measures derived from resting-state or task-related recordings. These features were predominantly employed for binary ASD versus neurotypical classification and consistently revealed atypical power distributions across canonical frequency bands, particularly involving alpha, beta, and gamma activity [54,55,56,57].

A second major research line investigated ASD-related alterations at the functional connectivity level, modeling interactions among EEG channels rather than isolated spectral features. Connectivity measures included coherence, phase-based synchronization indices, correlation-based metrics, mutual information, and dynamic connectivity representations computed across multiple frequency bands. Both resting-state and task-based paradigms highlighted atypical frontal, temporal, and long-range connectivity patterns in ASD [57,58,59,60,61,62,63]. While most studies framed connectivity features within diagnostic classification tasks, a limited subset explored alternative objectives, such as emotion recognition or symptom prediction, within autistic populations [64,65]. Closely related to functional connectivity approaches, several studies adopted structured representations of EEG data to explicitly encode spatial, temporal, or functional relationships among channels. These methods relied on graph-based, hypergraph, or graph signal processing formulations, from which topological or spectral descriptors were extracted and subsequently classified using conventional ML algorithms [17,58,59,60,66,67,68]. Although such representations enhanced the characterization of distributed network alterations in ASD, most studies did not explicitly quantify the contribution of individual nodes, edges, or substructures to model decisions.

Complementary research lines leveraged nonlinear and information-theoretic descriptors, such as sample entropy and multiscale entropy, often combined with adaptive signal decomposition techniques. These approaches aimed to characterize alterations in neural complexity and information flow, reporting reduced signal irregularity and atypical coupling patterns in ASD, particularly during task-based conditions [69,70,71]. Finally, a limited number of studies have explored multimodal ML frameworks by integrating EEG-derived features with complementary behavioral measures, such as eye-tracking data, to enhance diagnostic specificity and clinical relevance [72]. Despite their potential, such multimodal approaches remain relatively scarce and are predominantly based on handcrafted features and standard classifiers.

Overall, traditional ML approaches represent the methodological foundation of EEG-based AI research in ASD. These methods are generally based on handcrafted spectral, temporal, and connectivity-derived features that are informed by neurophysiological principles. While such strategies offer structured and physiologically interpretable representations of neural activity, their reliance on predefined feature extraction procedures and their limited capacity to learn complex hierarchical patterns have been associated with the increasing use of DL architectures and explicit XAI techniques in more recent studies.

#### 3.1.2. Hybrid and Shallow Neural Network-Based Methods

A limited number of EEG-based ASD studies have adopted shallow NNs and hybrid neural pipelines as an intermediate methodological paradigm between conventional ML and end-to-end DL approaches. These works relied on neural architectures with limited depth, including feedforward artificial NNs, self-organizing maps, cellular NNs, and neuro-fuzzy systems. Such models were typically embedded within multi-stage processing pipelines combining signal transformation, handcrafted feature extraction, feature selection, or optimization-based parameter tuning. In supervised settings, shallow and hybrid neural approaches were predominantly employed for binary classification between individuals with ASD and neurotypical controls. These models leveraged entropy-based, spectral, wavelet-derived, synchronization-related, or microstate-informed EEG features, rather than operating directly on raw signals [73,74,75,76,77,78,79,80]. Several studies reported high classification performance, particularly when nonlinear feature transformations, adaptive preprocessing strategies, or optimization-driven parameter tuning were applied. However, model interpretation was generally limited to post hoc statistical analyses of input features, channel-wise group comparisons, or qualitative inspection of intermediate representations, without explicit attribution of model decisions or formal explainability mechanisms. Beyond diagnostic classification, shallow and hybrid neural systems were also employed in non-predictive or exploratory settings to investigate latent EEG structure and network organization in ASD. For instance, neural architectures were used to estimate and compare effective connectivity patterns across groups, serving as data-driven modeling tools rather than classifiers [81]. Similarly, unsupervised shallow NNs were adopted for exploratory EEG analysis without predictive objectives, aiming to characterize intrinsic signal organization rather than to support classification or explanation [82]. In task-specific paradigms distinct from diagnostic classification, such as emotion recognition or affective processing, shallow and hybrid neural models were applied to decode task-related EEG responses within autistic populations [83]. In these cases, NNs functioned as task-oriented pattern recognition tools, with limited emphasis on interpretability beyond feature-level or descriptive analyses.

Overall, shallow and hybrid neural network approaches have contributed to the modeling of nonlinear relationships and structured EEG representations in ASD research. However, their interpretability was generally indirect, being primarily inherited from feature engineering strategies, signal transformations, or predefined network topologies rather than supported by dedicated XAI methodologies. In addition, many studies employing such models were conducted on relatively limited sample sizes and heterogeneous experimental protocols, often focusing on task-specific settings. These aspects have coincided with the increasing adoption of deeper neural architectures and the integration of formal XAI frameworks in more recent investigations, as discussed in the following sections.

#### 3.1.3. Deep Learning Methods

In recent years, DL methods have been increasingly adopted for EEG-based ASD analysis, driven by their ability to automatically learn hierarchical representations from high-dimensional and non-linear neurophysiological data.

A substantial portion of DL studies employed CNN-based architectures to model temporal and spatial EEG dynamics. These models were applied to raw EEG segments, time–frequency representations, or connectivity-derived maps, often achieving improved classification performance compared to conventional feature-based approaches. Several works further combined CNNs with recurrent units, such as long short-term memory (LSTM) or gated recurrent units, to capture temporal dependencies in dynamic functional connectivity or sequential EEG representations. Lightweight attention mechanisms were occasionally incorporated to enhance spatiotemporal modeling efficiency, particularly in real-time or resource-constrained settings [21,84,85,86,87]. Beyond Euclidean representations, a parallel research line explored graph-based DL models to explicitly encode the structure of EEG data. In these approaches, EEG channels or brain regions were modeled as nodes within graph structures, with graph convolutional networks (GCNs) and adaptive graph learning strategies employed to capture spatial dependencies, hemispheric asymmetries, and subject-specific functional connectivity patterns from resting-state EEG. Such graph-based DL frameworks demonstrated promising performance in ASD classification, highlighting the relevance of network-level representations in neurodevelopmental disorders [88,89]. More recent studies investigated deep representation learning paradigms, including contrastive and self-supervised learning, to derive discriminative EEG embeddings without explicit reliance on handcrafted features. These approaches aimed to improve robustness and generalization by leveraging intrinsic data structure and inter-subject variability, while still employing deep neural networks as the primary modeling backbone [90].

Despite their strong empirical performance, DL approaches for EEG-based ASD analysis are frequently characterized by limited intrinsic transparency. In a substantial portion of the reviewed studies, model outputs are not accompanied by formal attribution mechanisms capable of identifying the relative contribution of specific temporal segments, spectral components, channels, or connectivity patterns. Interpretability is therefore typically inferred indirectly from architectural design choices or post hoc statistical analyses. In parallel, recent studies have begun to incorporate dedicated XAI methodologies to provide more explicit explanations of model behavior [84,88].

### 3.2. Explainable AI in EEG-Based Studies for ASD

The increasing complexity of AI models applied to EEG analysis has emphasized the need for XAI frameworks capable of linking model decisions to physiologically meaningful neural patterns [37,38]. In EEG-based research, XAI plays a dual role: it enhances the transparency and trustworthiness of AI systems, while simultaneously enabling the interpretation of learned representations in relation to brain function and dysfunction. From an operational perspective, XAI aims to identify which aspects of the EEG signal, across temporal, spectral, spatial, or connectivity dimensions, drive model predictions, thereby supporting neurophysiological interpretation rather than solely algorithmic validation.

AI methods can be classified along two conceptually independent dimensions: whether interpretability is intrinsic to the model or introduced through post hoc explanation techniques, and whether the method is model-specific or model-agnostic. Inherently interpretable approaches rely on model architectures whose internal mechanisms are directly accessible and semantically meaningful. In such models, parameters maintain an explicit relationship with input features, allowing direct inspection of how predictions are generated. Typical examples include linear models and decision trees, where coefficients or decision rules can be examined without additional explanatory procedures. In EEG research, interpretability may also be supported by structured transformations that recover physiologically meaningful activation patterns from model parameters, such as the method proposed by Haufe et al. [91], or by constrained architectures designed to preserve correspondence with neurophysiological features [92]. Inherently interpretable models are necessarily model-specific, as their transparency depends on their internal structure. By contrast, post hoc explanation techniques are applied after model training and aim to clarify how a predictive model produces its outputs. These methods analyze input–output relationships to estimate the contribution of specific features to predictions. Post hoc techniques may be model-specific, when they require access to internal model components (e.g., gradient-based saliency methods for neural networks), or model-agnostic, when they treat the predictive model as a black box and rely solely on probing its input–output behavior. Examples of model-agnostic approaches include surrogate models such as LIME and feature attribution frameworks such as SHAP [39,40,93]. While post hoc methods offer broad flexibility, they may introduce approximation errors or attribution instability, particularly in high-dimensional and noisy domains such as EEG. Consequently, their application requires careful methodological validation and transparent reporting.

Within EEG analysis, XAI can further operate at different levels of signal representation. Feature-based approaches assess the relevance of predefined EEG descriptors, such as spectral power, functional connectivity metrics, or graph-theoretical measures, thereby facilitating direct neurophysiological interpretation. Attribution-based approaches, instead, act on raw or minimally processed signals to identify salient temporal segments, frequency bands, channels, or network components that contribute most strongly to model predictions [94,95]. These different levels of explanation determine how XAI outputs can be mapped onto clinically interpretable constructs, such as oscillatory dynamics, regional brain activity, or large-scale network organization.

Crucially, meaningful explainability in EEG-based AI extends beyond technical transparency and requires alignment with established neurophysiological knowledge. Explanations should preserve spatial, temporal, and spectral coherence, demonstrate consistency across subjects or populations, and enable a principled mapping between algorithmic relevance patterns and known models of brain organization [96,97]. These requirements are particularly relevant in neurodevelopmental disorders such as ASD, where clinical heterogeneity and complex neural dynamics challenge the transparency and generalization of purely performance-driven models [98,99].

Despite the growing body of AI-based EEG research in ASD, many studies continue to prioritize predictive performance over interpretability and systematic explainability, often relying on black-box models that provide limited insight into the neurophysiological mechanisms underlying classification or regression outcomes [100,101]. This lack of transparency represents a major obstacle to clinical translation, where model trust, validation, and neurobiological plausibility are essential.

For these reasons, the present systematic review focuses specifically on studies that explicitly incorporate explainability or interpretability components into AI-based EEG analysis of ASD. Section 4 synthesizes how different XAI paradigms have been applied, the levels at which explanations are generated, and the neurophysiological insights enabled across the included studies.

## 4. Results

### 4.1. General Characteristics of the Included Studies

The eleven studies included in this review were published between 2020 and 2025 and were conducted across countries with diverse cultural and research contexts, including the United States [32,50,52], Poland [49], Belgium [49], United Kingdom [47,48], China [53], India [45,46,47,48], Italy [44,50], Romania [51], Sweden [51], and Portugal [51,102]. Despite the heterogeneity of the cultural settings, most of the studies were carried out in academic institutions and university departments, in collaboration with hospitals and clinical centers specialized in neurodevelopmental disorders. Eight studies were designed as computational investigations, in which previously collected EEG data were used exclusively for model training and algorithmic analysis rather than to evaluate the effects of clinical interventions [44,46,47,48,49,50,51,53]. In addition, the reviewed literature included one prospective longitudinal observational study [32], one cross-sectional case–control study [45], and one methodological study focusing on model interpretability and validation [52]. All studies investigated ASD and included participants diagnosed with ASD or considered at elevated risk for the condition. In the two studies involving infant cohorts [32,46], participants had not received a formal ASD diagnosis due to their young age; instead, ASD risk was inferred based on familial history or early neurophysiological indicators. Notably, Irram et al. [46] also examined EEG data from infants identified as being at risk for other neurodevelopmental conditions, such as language or learning difficulties, without subsequent diagnostic confirmation. In contrast, in the longitudinal study by Dickinson et al. [32], ASD diagnoses were confirmed in 14 participants during follow-up. With the exception of the study by Borra et al. [44], all investigations included a control group of TD individuals, matched for age with the ASD or at-risk cohorts. EEG recordings were predominantly acquired under resting-state conditions, with eyes open or closed, or during passive exposure to low-demand neutral stimuli, such as soap bubbles [52], to enhance participant compliance and minimize motion-related artifacts. One study analyzed EEG data collected during sleep as part of routine clinical practice [49]. In contrast, five studies employed task-based paradigms, including auditory or visuoperceptual tasks, facial emotion recognition, cartoon viewing, or ADOS-2-related procedures [44,45,48,50,51].

The included studies also exhibited substantial heterogeneity in EEG acquisition protocols, particularly with respect to channel density, which ranged from 3 to 129 electrodes [32,45]. This variability reflects the limited standardization of EEG-based methodologies in XAI-oriented research and highlights the ongoing gap between routine clinical EEG practice and the systematic integration of advanced XAI frameworks in neurodevelopmental research.

A summary of the main characteristics of the included studies is provided in Table 3.

#### 4.1.1. Sample Characteristics

The included articles exhibit a substantial heterogeneity in terms of the involved samples and, in numerous cases, information is partial or incomplete. The first relevant difference, which has also a significant impact on the interpretation of the results, concerns sample size. Specifically, the sample size varies considerably across studies, ranging from 15 participants [44] to 293 in the study by Carson et al. [52]. Similarly, the age represents another source of heterogeneity across studies, as it ranges between 3 months [32] and 68 years of age [53]. In particular, two articles analyzed EEG data collected from infants in first months of life [32,46], five considered children in early childhood and in school age [45,48,49,51,52], whereas Shi et al. [53] analyzed data belonging to a mixed sample, made of children, adolescents and adults. In two cases, authors did not express age range, but only mean age (respectively 15 years and 22 years) [44,50]. Sex distribution was inconsistently reported and described across the studies too. In six papers [32,47,48,49,50,52], the samples consist of a marked male predominance, according to the well-known epidemiological ratio of ASD [102,103], with a very small proportion of females diagnosed with ASD. Furthermore, socio-economic status and ethnicity were rarely reported, except for the study by Dickinson et al. [32]. In this case, the sample comprised 58% of White participants, 25% of people identifying with more than one race and 16.7% from ethnic minority groups (Asian, Black and Pacific Islander). Additionally, 44.4% of the sample was Hispanic and 77.8% of mothers held a college degree.

#### 4.1.2. ASD Clinical Profile

Considering the ASD samples involved in these eleven articles, cognitive functioning varies across studies. In most articles, cognitive functioning was within the typical range, whereas in two studies participants exhibited low developmental quotients and cognitive impairments [32,52]; notably, five studies did not report IQ or cognitive functioning, limiting sample characterization and generalizability of findings to the entire spectrum of ASD. Little information was provided regarding severity of the disorder and the level of expressive abilities, as well as regarding comorbidities and pharmacological medications. Based on these latter aspects, only the study by Carson et al. [52] reported more details. Comorbidities were explicitly reported in only one study in terms of psychomotor delay [49], while two studies used comorbidity as an exclusion criteria. Similarly, stimulant use was documented in only one study, whereas pharmacological treatment [52] constituted an exclusion criterion in another article [51]. The lack of information about comorbidities likely reflects the use of strict exclusion criteria in EEG research.

A summary of all relevant information is presented in Table 4.

### 4.2. XAI Methods Applied to EEG-Based ASD Studies

To facilitate interpretation of the findings, the included studies were grouped according to the underlying AI paradigm and the corresponding XAI strategies adopted. Specifically, results are organized into explainable ML (ML-XAI) and explainable DL (DL-XAI) approaches, reflecting fundamental differences in model structure and feature representation. This conceptual framework enables a systematic comparison of how interpretability and explainability are conceptualized and implemented across methodological paradigms, and how resulting explanatory outputs relate to neurophysiological features, functional connectivity patterns, and clinically relevant EEG markers in ASD.

#### 4.2.1. Explainable Machine Learning Approaches

A substantial portion of XAI studies on EEG-based ASD analysis relies on classical ML paradigms rather than DL architectures. This preference reflects the explicit separation between feature extraction and model learning inherent to traditional ML pipelines, which facilitates the direct interpretability of learned associations and the incorporation of neurophysiological priors. In ASD research, where datasets are often limited and clinical understanding is critical, ML-XAI frameworks offer a pragmatic balance between predictive modeling and scientific insight.

Across the reviewed literature, ML-XAI approaches were applied to both binary diagnostic classification and dimensional prediction of ASD symptom severity. Linear and kernel-based models, including logistic regression, SVMs, and support vector regression, were predominantly employed [45,46,47,49]. Regression-based formulations were adopted in a subset of studies to preserve continuous relationships between EEG-derived features and behavioral outcomes, enabling more nuanced interpretation beyond categorical diagnosis [32]. Explainability within ML-XAI frameworks was achieved through a combination of model-specific and post hoc approaches. Linear models enabled intrinsic feature attribution through direct inspection of model coefficients, while transformations such as the Haufe method were used to recover physiologically meaningful activation patterns from decoding weights [32,52]. In parallel, model-agnostic techniques, most notably SHAP and ELI5, quantified global and local feature contributions across different classifiers while accounting for feature dependencies [45,46,47,49]. Beyond methodological diversity, ML-XAI studies reported convergent explanatory findings. At the connectivity level, XAI analyses consistently identified altered long-range functional connectivity as a key driver of model predictions, with reduced frontal integration emerging as a robust explanatory pattern associated with ASD diagnosis or increased symptom severity across pediatric and infant cohorts [32,47,49]. In early infancy, increased right temporoparietal connectivity, particularly alpha-band coherence at three months of age, was shown to predict higher symptom severity at later developmental stages [32]. At the spectral level, ML-XAI approaches highlighted the relevance of specific frequency bands in a development-dependent manner. Alpha-band features were primarily associated with early functional connectivity and network maturation, whereas gamma-band activity emerged as a discriminative component in older cohorts [45,52]. Importantly, several studies demonstrated that features yielding high predictive performance did not necessarily correspond to stable or reproducible explanatory patterns, underscoring the distinction between performance-driven and XAI-driven modeling strategies [52]. From a methodological perspective, ML-XAI studies showed that the stability and neurophysiological plausibility of explanations strongly depended on model selection and regularization strategies. Frameworks explicitly optimized for interpretability and textcolorredexplainability produced more consistent patterns across cross-validation splits, whereas purely performance-oriented optimization often resulted in unstable or heterogeneous feature attributions [52]. These findings indicate that XAI is not an automatic consequence of using simple models but the result of deliberate methodological design aimed at extracting reliable EEG–ASD associations.

Overall, ML-XAI approaches represent one of the most methodologically mature and conceptually grounded form of XAI currently applied to EEG-based autism research. By enabling multilevel interpretation of spectral, spatial, and network-level features, these frameworks have provided reproducible insights into ASD-related neural alterations, particularly in frontal and temporoparietal connectivity [32,47,49,52]. However, their reliance on handcrafted features and relatively shallow models limits their capacity to capture complex non-linear and spatiotemporal EEG dynamics, motivating the growing adoption of DL architectures and associated explainability frameworks discussed in the following section.

#### 4.2.2. Explainable Deep Learning Approaches

In recent years, DL architectures have been increasingly adopted for EEG-based ASD analysis due to their ability to model complex, non-linear, and high-dimensional signal representations. Convolutional, recurrent, graph-based, and hybrid deep networks have demonstrated strong predictive performance across both resting-state and task-related EEG paradigms [84,88]. However, the intrinsic opacity of deep models poses substantial challenges for clinical adoption and neuroscientific interpretation, motivating the integration of XAI techniques within DL frameworks [42].

Across the reviewed literature, DL-XAI approaches were applied predominantly to binary ASD classification tasks [44,48,51,53], with a smaller number of studies addressing non-diagnostic objectives, such as emotion recognition [50]. Model architectures varied widely, ranging from constrained convolutional networks designed to capture specific electrophysiological components to multi-stream and graph-based models integrating temporal, spectral, and spatial information [44,48,53].

In DL models, explainability was achieved primarily through post hoc explanation techniques, although a limited number of studies incorporated elements of inherent interpretability within the network architecture. In these cases, architectural constraints were introduced to preserve the correspondence between learned representations and physiologically meaningful EEG components, for example by designing convolutional filters to capture event-related potentials [44]. Such approaches aim to embed interpretability directly within the model structure and are therefore intrinsically model-specific. More commonly, post hoc explanation methods were applied after model training to analyze prediction mechanisms. These included model-specific techniques, such as gradient-based saliency maps, class activation mapping, Integrated Gradients, and attention weight analysis, which rely on access to internal network parameters. In addition, model-agnostic approaches such as SHAP were employed to estimate feature contributions by probing the input–output behavior of the trained model [48,51,53]. These post hoc strategies were used to identify salient temporal segments, frequency bands, EEG channels, or graph elements contributing to model predictions. Despite methodological heterogeneity, DL-XAI studies reported convergent explanatory patterns. At the temporal level, explainability analyses consistently highlighted atypical processing of salient EEG components in ASD, including delayed or attenuated task-related responses such as the P300 [44]. When interpretability was incorporated by design, convolutional filters captured reduced ERP amplitude and prolonged latency, with salient activations localized over centro-parietal regions, in agreement with established electrophysiological findings. At the spectral and spatial levels, post hoc DL-XAI analyses frequently emphasized the contribution of low-frequency activity, particularly in the theta and alpha bands, alongside region-specific patterns involving frontal, central, and parietal electrodes. Several studies reported reduced frontal and fronto-parietal relevance for neurotypical classifications and increased emphasis on central or temporoparietal regions for ASD predictions [51,53]. Some studies further extended DL-XAI to graph-based representations, applying attribution methods to graph convolutional networks operating on EEG-derived graphs. These approaches enabled node- and edge-level relevance visualization, offering insights into hierarchical or topological signal organization [48]. However, the resulting explanations often reflected abstract graph structures and remained only indirectly interpretable in terms of canonical neurophysiological constructs, such as functional connectivity between brain regions. A critical limitation emerging from DL-XAI studies concerns the stability and reliability of explanations. Comparative analyses showed that high predictive accuracy does not necessarily correspond to robust or consistent explanatory patterns across subjects or perturbations. Attribution maps were frequently sensitive to model initialization, architectural choices, and input variability, raising concerns about the overinterpretation of visually compelling explanations [50,53].

Overall, DL-XAI approaches have expanded EEG-based ASD research by enabling the analysis of rich spatiotemporal representations beyond the reach of classical ML. When interpretability is embedded into model design, deep networks can recover physiologically meaningful electrophysiological markers. Nevertheless, the prevalent reliance on post hoc attribution techniques yields explanations that are often descriptive rather than mechanistic. This tension between predictive performance and explanatory robustness underscores the need for rigorous validation, stability assessment, and stronger neurophysiological grounding before DL-XAI frameworks can be reliably adopted for clinical or scientific inference [44,50,53]. In practical terms, explanation robustness can be implemented through minimal validation procedures that assess the consistency of attribution patterns under controlled variations. For instance, explanations may be compared across different random seeds or cross-validation splits to evaluate stability with respect to model initialization and data partitioning. Perturbation or occlusion analyses can be used to verify whether features identified as relevant genuinely influence model predictions when systematically altered. Additionally, agreement across multiple attribution methods applied to the same trained model may provide complementary evidence regarding the reliability of identified neurophysiological patterns. Treating explanation stability as an empirical outcome, rather than an implicit assumption, may help establish minimal methodological standards for future EEG-XAI research in ASD.

Table 5 provides a summary of the relevant information.

### 4.3. Interpretable Electrophysiological Findings in ASD

Across the eleven studies included in this systematic review, electrophysiological differences between individuals with ASD and TD controls were examined using spectral, temporal, and functional connectivity features extracted from EEG recordings. Seven studies focused on resting-state spectral and connectivity alterations (of which one used data collected from sleeping children) [51], whereas four investigated spatial and temporal neural markers during task-based paradigms, including P300 decoding and facial emotion recognition.

A summary of all relevant information is presented in Table 6.

#### 4.3.1. Spectral Alterations

Resting-state spectral analyses consistently revealed atypical frequency-band activity in ASD across multiple developmental stages. Several studies reported that low-frequency activity (5–10 Hz; theta–alpha range) was more strongly associated with TD classification, whereas increased gamma-band activity (>40 Hz), particularly in terms of power and functional connectivity, was associated with ASD diagnosis, predominantly over occipital and posterior midline regions [52]. In children with ASD, significantly higher spectral power across theta, alpha, low beta, and high beta bands was observed over frontal and central electrodes compared to TD peers. Moreover, while TD individuals exhibited a typical anterior–posterior power gradient, ASD participants showed a more spatially uniform distribution of spectral power [49]. In the study by Shargilavan et al. [51], the ASD population exhibited increased delta and theta activity, rather than they typically exhibited reduction in alpha activity. These electrophysiological atypicalities may reflect neuronal immaturity and alterations in cortical inhibitory mechanisms. In particular, high theta activity in fronto-central regions may be associated with impairments in attentional control and self-regulation, while reduced alpha activity has been linked to altered sensory processing, including sensory hyper-reactivity, as well as attentional dysregulation [104]. In infant cohorts, ML models identified elevated delta and gamma power within left frontal regions as predictive of later ASD diagnosis [46]. Additionally, one study reported that the 12–16 Hz frequency range (overlapping high alpha and low beta bands) provided optimal discrimination between ASD and TD groups [45]. Gamma oscillations appear to play a key role in cortical excitation and visual attention [27,28,105,106,107] whereas alpha contributes in regulation and inhibition processes [23,26,108]. Increased gamma activity and reduced alpha activity are consistent with the hypothesis of an impaired excitation/inhibition balance in autistic individuals, which are likely associated to GABA-related neurotransmitter systems [23,26,108,109,110,111]. According to the literature, this imbalance may reflect differences in visual attentional behaviors [112,113,114].

#### 4.3.2. Functional Connectivity and Network Organization

Functional connectivity analyses highlighted atypical network organization in ASD, characterized by altered integration and hierarchical structure [47,49]. Weighted Hierarchical Complexity (WHC) analyses demonstrated reduced hierarchical depth and complexity in ASD networks compared to TD networks, with ASD networks showing stronger local connectivity and weaker long-range connections [47]. These aspects are linked to reduced high-level functional integration and globally inefficient connectivity, and may reflect poor complex behavioral performance and intellectual capacity [71,115]. In addition, the Visible-Graph Convolutional Network (VGCN) identified an imbalance in neuronal hierarchy between primary sensory regions and higher-order associative areas, potentially compromising the segregation of cognitive processes [8]. Phase-based connectivity results depended on the metric employed. While Phase Locking Value (PLV) analyses indicated increased connectivity in ASD—particularly between frontal and centro-parietal regions—corrected imaginary PLV (ciPLV) revealed reduced non-zero-lag connectivity in ASD, especially within the low beta band [49]. Moreover, coherence analyses identified a poorly synchronized functional connectivity in ASD. Individuals with ASD exhibited reduced long-range coherence in Delta–Theta and Theta–Alpha bands, mostly affecting fronto-parietal and fronto-temporal networks. This reduced coherence may underlie difficulties in executive control, attentional and social modulation and language. By contrast, short-range connectivity mediated by gamma bands exhibited hyper-synchronization reflecting reduced flexibility and focused processing style [51]. Longitudinal analyses in infants further showed that reduced frontal alpha-band coherence and increased right temporoparietal coherence at 3 months of age were associated with higher ASD symptom severity at 18 months [32]. The right temporo-parietal junction is considered a “social hub” involved in social information processing [116], and it shows atypical functioning in ASD [117]. Increased alpha-band coherence may reflect a network inefficiency and early hypoconnectivity that potentially contribute to the hub overload [118].

#### 4.3.3. Task-Based Spatial and Temporal Differences

Task-related EEG analyses revealed group-specific differences in both the spatial localization and temporal dynamics of neural responses [44,50]. During P300 paradigms, DL-XAI approaches identified a pronounced right-parietal asymmetry in ASD participants, with model-derived features showing strong correlations with ADOS-2 clinical scores, unlike conventional ERP measures [44]. These findings align with the previous literature suggesting right-hemispheric lateralization for social perception, especially in the processing of facial expressions and gaze orientation [64,119,120,121]. Moreover, the P300 component is implicated in several cognitive processes [122,123], including attention and working memory, which are often compromised in ASD [124,125,126,127,128]. In facial emotion recognition tasks, XAI analyses demonstrated that ASD-TD differences were most prominent during later processing stages (approximately 500–1500 ms post-stimulus), particularly for negative emotions. Temporal relevance patterns indicated that ASD participants relied on additional processing stages compared to TD individuals, suggesting altered temporal dynamics of emotional information processing [50]. Finally, age-dependent interpretability analyses revealed that the spatial features most relevant for ASD classification varied across development, with occipital regions contributing most strongly in adults and prefrontal regions showing greater relevance in adolescents [53]. These atypical patterns align with findings reporting altered visual processing and the integration of visual stimuli, as well as impairments in executive-functions in ASD [129,130,131].

## 5. Discussion

The concurrent increase in the prevalence of neurodevelopmental disorders and the rapid advancement of technological tools have driven the growing adoption of sensitive and minimally invasive techniques to characterize these conditions and investigate their developmental trajectories. In this context, EEG has become a widely used technique in clinical practice. Both resting-state and task-based EEG analyses have enabled the identification of common and disorder-specific neurofunctional characteristics, which are increasingly considered objective markers of atypical brain functioning. The application of AI technologies has further enhanced EEG signal analysis by enabling the detection of complex patterns that are not readily identifiable through traditional analytical approaches or clinical inspection alone. However, the limited transparency of many AI models represents a critical barrier to their clinical adoption, particularly in neurodevelopmental research, where neurophysiological plausibility is essential. In this context, XAI approaches offer a substantial added value by providing insights into the electrophysiological features, temporal dynamics, and functional interactions that drive model predictions. By bridging the gap between predictive performance and mechanistic understanding, XAI frameworks facilitate the translation of AI-based EEG findings into clinically meaningful knowledge, supporting hypothesis generation, diagnostic decision-making, and the development of personalized and preventive intervention strategies. Accordingly, the present review systematically collected and analyzed the available literature on XAI methods applied to the study of electrophysiological correlates in populations with ASD, identifying a total of 11 eligible studies. From a methodological perspective, the reviewed literature predominantly focused on EEG data acquired during resting-state conditions, typically involving eyes-open or eyes-closed relaxation paradigms. To facilitate compliance, especially in pediatric populations, some studies collected EEG recordings during exposure to low-demand, relaxing stimuli such as soap bubbles [52].

Only a limited number of studies [44,45,48,50,51] employed task-based paradigms, indicating a prevailing emphasis on the investigation of spontaneous brain activity. Resting-state experimental designs are particularly suitable for pediatric and clinically vulnerable populations, including individuals with adaptive difficulties, as they allow the examination of intrinsic neural dynamics and functional connectivity networks with minimal task demands. Furthermore, resting-state paradigms are generally more reproducible over time and less susceptible to confounding sources of variability, thereby facilitating the construction of large and comparable datasets.

Across the included studies, EEG data were acquired using both high- and low-density systems, with the number of electrodes ranging from 4 to 129. Analyses primarily focused on specific regions of interest, particularly frontal and central areas, where abnormalities in functional connectivity and spectral organization have been consistently reported [132]. Overall, the 11 studies identified convergent electrophysiological differences between individuals with ASD and TD controls across spectral, temporal, and functional connectivity domains. These differences enabled robust discrimination between ASD and TD groups and supported neuropsychological interpretations of ASD-related brain functioning. Notably, an imbalance between alpha and gamma oscillations emerged as a consistent finding. In line with previous literature, individuals with ASD exhibited increased gamma-band activity and reduced alpha power compared to TD controls [49,51,52]. Such spectral alterations are consistent with sensory processing abnormalities, including hyper- or hyporeactivity to environmental stimuli, as well as attentional regulation difficulties frequently observed in ASD.

At the neurophysiological level, atypical gamma and alpha oscillatory activity has been interpreted as potentially reflecting a disruption in cortical excitation–inhibition balance, likely associated with alterations in neurotransmitter systems, particularly GABAergic circuits [22,26,108]. These findings further supported the view of ASD as a complex, multicomponent, and fundamentally neurobiological condition. In addition to spectral alterations, several studies highlighted atypical patterns of neural connectivity in ASD populations. These connectivity abnormalities may play a critical role in distinguishing ASD from TD individuals and are characterized by simplified hierarchical network structures, with a predominance of short-range connections at the expense of long-range integration [47]. Reduced global efficiency and functional integration appear to be hallmark features of ASD-related brain networks and may contribute to the cognitive and social impairments commonly associated with the condition [8,71,115,116,117,118]. Importantly, altered alpha-band coherence has been shown to predict symptom severity at later developmental stages, highlighting its potential prognostic value [32].

Task-based studies further may contribute to corroborate differences in neural functioning between ASD and TD individuals, revealing right-hemispheric asymmetries involving regions implicated in attentional modulation and social stimulus processing [44,50,64,119,120,121,124,125,126,127,128]. Within this context, XAI techniques such as SHAP, LRP, and ELI5 demonstrated particular promise in identifying objective spectral, spatial, and temporal features relevant for ASD classification and in providing clinically meaningful interpretations of model decisions [44]. The reviewed studies illustrated the potential of XAI approaches to identify novel biomarkers, validate existing neuroscientific hypotheses, and address clinical heterogeneity. For example, XAI methods enabled the detection of associations between ERP and clinical severity scores measured using the ADOS-2, which are not readily identifiable using conventional analytical techniques [44]. Additionally, these approaches facilitated the evaluation of feature relevance in distinguishing between clinical conditions [50] and supported the identification of potential clinical subgroups characterized by distinct electrophysiological patterns [49].

Importantly, these interpretative advances cannot be attributed to model architecture alone, but rather to the interaction between modeling strategies, data representations, and the explainability frameworks adopted across studies. Although most reviewed approaches relied on post hoc explanation techniques, these methods differed in their degree of model dependence. Model-specific approaches, such as Layer-wise Relevance Propagation (LRP) and gradient-based saliency methods, required access to internal network parameters, whereas model-agnostic techniques, including certain implementations of SHAP, operated by probing the input–output behavior of the trained model [38,42]. Regardless of this distinction, the stability and neurophysiological plausibility of the resulting explanations were strongly influenced by feature representation and model constraints. ML frameworks operating on explicitly defined spectral or functional connectivity features tended to produce more stable and physiologically interpretable explanations, as their representations preserved a direct correspondence with EEG markers. In contrast, DL approaches, while capable of capturing richer spatiotemporal dependencies, often generated attribution patterns that were more sensitive to architectural design choices and input variability. Overall, these findings indicate that explainability in EEG-based ASD research depends not only on the choice of explanatory technique, but also on how that technique interacts with model structure and data representation [50].

Despite these findings, the application of XAI approaches to EEG analysis in ASD remains in an early and evolving stage. The innovative nature of XAI methodologies is reflected in the substantial heterogeneity and limited systematicity observed across experimental designs. Many studies provided only partial descriptions of sample characteristics, with insufficient clinical characterization of ASD developmental profiles. Key parameters—including symptom severity, cognitive and verbal functioning, and, in some cases, participant age range—were frequently absent, imprecise, or not integrated into EEG analyses. These limitations affect both resting-state and task-based studies and hinder the identification of clinically meaningful subgroups and the prediction of developmental trajectories. This issue is particularly relevant given the high rate of comorbidity in ASD, including conditions such as attention-deficit/hyperactivity disorder (ADHD), which is itself associated with electrophysiological alterations [133,134]. Such comorbidities contribute additional sources of variability and complicate the attribution of observed EEG abnormalities specifically to ASD [135,136].

Another important source of heterogeneity concerns the EEG acquisition systems and channel density employed across studies. While some variability is expected in emerging research fields, these methodological differences may limit the robustness, comparability, and generalizability of findings. As XAI-based EEG research continues to expand, it is anticipated that the growing diffusion of these approaches will promote the adoption of more rigorous, transparent, and reproducible experimental designs. Substantial methodological consolidation and rigorous validation will be essential before XAI-based approaches could be considered reliable in clinical-decision making procedures.

In addition, a further limitation concerns the definition-based inclusion boundary adopted in this review. By restricting eligibility to studies that explicitly framed their interpretability strategies within an XAI paradigm, we may have excluded investigations implementing functionally similar interpretative analyses without adopting explicit XAI terminology. This choice reflects the methodological focus of the review on the development and application of XAI as a defined framework, rather than on all forms of model interpretability in EEG-based ASD research. Consequently, the findings should be interpreted as mapping the current state of explicit XAI applications in this field. Taken together, these considerations highlight the need for clearer methodological standards to support the maturation of the field. In light of the evidence summarized in this review, a set of practical recommendations can be outlined to support the translation of XAI-based EEG approaches into clinically meaningful applications. These recommendations directly address the methodological and interpretative challenges identified across the reviewed studies and aim to provide concrete guidance for future research. First, external validation and cross-dataset evaluation should be prioritized whenever feasible. XAI-based EEG models should be tested on independent cohorts or across datasets acquired at different sites to assess generalizability and to reduce the risk of dataset-specific patterns or explanations. When external datasets are not available, cross-dataset or leave-site-out validation strategies should be considered as minimal requirements. Second, the stability and reproducibility of explanations should be treated as explicit outcome measures. Beyond predictive performance, future studies should systematically assess the robustness of XAI outputs across subjects, recording sessions, model initializations, and perturbations of the input data. Both quantitative metrics and qualitative evaluations of explanation consistency should be reported, as unstable or highly variable explanations limit clinical trust even in the presence of high classification accuracy. Third, XAI outputs should be explicitly linked to clinically relevant phenotypes. To enhance clinical interpretability, relevance patterns derived from XAI analyses should be related to symptom severity, developmental trajectories, cognitive or behavioral profiles, and task performance measures when available. Establishing such links is essential to move from descriptive model explanations toward actionable neurophysiological markers that can inform diagnosis, prognosis, and personalized intervention strategies.

Together, these steps provide a concise and operational roadmap for guiding future XAI-based EEG research toward robust, interpretable, and clinically translatable applications in ASD. Within this framework, minimal methodological expectations can also be articulated. Future studies applying XAI to EEG in ASD should demonstrate at least one form of robustness analysis and, whenever feasible, one form of external validation. Explanation plausibility alone, without empirical assessment of stability and generalizability, is insufficient to support clinical translation. By embedding these minimal standards into study design and reporting practices, the field may progressively move from visually compelling interpretations toward reproducible and clinically grounded explanatory frameworks.

## 6. Conclusions

This systematic review aimed to collect and analyze the current literature on the application of XAI technologies to the analysis of electrophysiological measures in populations with ASD. Overall, the findings highlight the substantial potential of XAI-based frameworks to advance EEG-driven neuroscience research and to support future clinical translation. In particular, the integration of EEG and XAI provides a non-invasive and interpretable approach for identifying novel neurophysiological patterns associated with neurodevelopmental disorders, validating previously reported electrophysiological features by quantifying their discriminative contribution relative to neurotypical populations, and detecting potential early biomarkers. Such biomarkers may be especially valuable during early developmental stages or in clinical contexts in which conventional diagnostic procedures are difficult to apply or lack sufficient sensitivity.

Beyond predictive performance, XAI methodologies enable the attribution of model decisions to specific EEG features across spectral, temporal, spatial, and connectivity-related dimensions. This capability facilitates a direct link between algorithmic outputs and underlying neurophysiological mechanisms and represents a key advantage over purely black-box AI models. As such, XAI is essential for enhancing understanding, trust, and applicability in clinical and translational settings. Based on the evidence summarized in this review, future research should prioritize larger and more comprehensively characterized clinical cohorts, together with rigorous evaluation of explanation stability, reproducibility, and neurophysiological plausibility. In addition, greater attention to developmental trajectories, cognitive profiles, and clinical heterogeneity will be crucial for refining neurodevelopmental subtyping within ASD and for guiding the development of reliable, interpretable, and clinically meaningful AI-based EEG tools.

## Figures and Tables

**Table 1:** Search strategy.

Database	Search String
PubMed	((“EEG” OR “Electroencephalogram” OR “Electroencephalography” OR “ERP”) AND (“Explainability” OR “XAI” OR “Interpretability” OR “Explainable” OR “Explain” OR “Connectivity”) AND (“ASD” OR “autism” OR “autism spectrum disorder”) AND (“AI” OR “Artificial Intelligence” OR “Deep Learning” OR “Machine Learning”))
Scopus	(KEY(“EEG” OR “Electroencephalogram” OR “Electroencephalography” OR “ERP”) AND KEY(“Explainability” OR “XAI” OR “Interpretability” OR “Explainable” OR “Explain” OR “Connectivity”) AND KEY(“autism” OR “autism spectrum disorder” OR “ASD”) AND KEY(“Artificial Intelligence” OR “AI” OR “Deep Learning” OR “Machine Learning”))
EBSCO Databases	(APA PsycArticles, APA PsycInfo, MEDLINE, CINAHL Plus with Full Text, Psychology and Behavioral Sciences Collection) (“EEG” OR “electroencephalogram” OR “electroencephalography” OR “ERP”) AND (“Explainability” OR “XAI” OR “Interpretability” OR “Explainable” OR “Explain” OR “Connectivity”) AND (“ASD” OR “autism” OR “autism spectrum disorder”) AND (“AI” OR “Artificial Intelligence” OR “Deep Learning” OR “Machine Learning”)
Web Of Science	(ALL=(“EEG” OR “Electroencephalogram” OR “Electroencephalography” OR “ERP”) AND ALL=(“Explainability” OR “XAI” OR “Interpretability” OR “Explainable” OR “Explain” OR “Connectivity”) AND ALL=(“autism” OR “autism spectrum disorder” OR “ASD”) AND ALL=(“Artificial Intelligence” OR “AI” OR “Deep Learning” OR “Machine Learning”))
Cochrane Library	(“autism spectrum disorder” OR autism OR ASD) in Title/Abstract/Keyword AND (“electroencephalogram” OR electroencephalography OR EEG OR ERP) in All Text AND (“artificial intelligence” OR AI OR “deep learning” OR “machine learning”) in All Text AND (explainability OR XAI OR interpretability OR explain OR connectivity) in All Text
IEEE Xplore	(“All Metadata”:“EEG” OR “All Metadata”:“Electroencephalogram” OR “All Metadata”:“Electroencephalography” OR “All Metadata”:“ERP”) AND (“All Metadata”:“Artificial Intelligence” OR “All Metadata”:“AI” OR “All Metadata”:“Deep Learning” OR “All Metadata”:“Machine Learning”) AND (“All Metadata”:“ASD” OR “All Metadata”:“autism” OR “All Metadata”:“autism spectrum disorder”) AND (“All Metadata”:“Explainability” OR “All Metadata”:“XAI” OR “All Metadata”:”Interpretability“ OR ”All Metadata“:”Explainable“ OR ”All Metadata“:”Explain“ OR ”All Metadata“:”Connectivity“)

**Table 2:** Methodological synthesis and explainable AI assessment across the included studies.

Study (Author, Year)	ASD and Control Diagnosis	EEG Preprocessing	Validation (CV, Hold-Out)	Imbalance and Confound Control	XAI Evaluation (Method, Consistency, Examples)	Code/Data Availability
Borra et al. (2022) [44]	ADOS, ADI-R; 15 ASD adults	2–30 Hz; notch 50 Hz; downsampled to 128 Hz	Nested CV (80/20) + hold-out test	Class weighting (M0/M1 = 7)	Gradient saliency: ADOS correlation (r = 0.801); right parietal (P4) asymmetry	Kaggle
Saranya & Menaka (2025) [45]	DSM-5, ISAA; 10 ASD, 10 TD (5–7 yrs)	Wavelet (db4); Butterworth 0.53–70 Hz	10-fold CV (80/20 split)	Numerically balanced groups	SHAP: peak-to-peak amplitude as most influential feature	Ethical protocol reported
Irram & Suaib (2025) [46]	Familial risk (HL/LL); 281 infants	1–45 Hz FIR; ICA (RUNICA); ICLabel	Stratified k-fold CV (80% training)	SMOTE applied to balance ASD class	SHAP: left frontal delta power strongest ASD predictor	Zenodo
Wadhera & Mahmud (2022a) [47]	DSM-5, ICD-10; 3 datasets (children/adults)	0.1–40 Hz; ICA (EEGLAB); visual inspection	Repeated 10-fold CV (20×); 70/30 split	Chi-square tests excluded age/sex/IQ bias	ELI5: WHC reveals reduced neural hierarchy in ASD	Available upon request
Wadhera & Mahmud (2022b) [48]	DSM-5, ADOS-2; heterogeneous datasets	0.1–40 Hz; ICA; ocular artifact removal	Repeated 10-fold CV (20×); 70/30 split	Dataset mixing to address heterogeneity	Integrated Gradients: visualization of critical nodes and edges	Available upon request
Rogala et al. (2023) [49]	DSM-5, ICD-10; 18 ASD, 18 TD (2–6 yrs)	1–45 Hz; re-referenced to Fz; sleep EEG	Nested CV (6 outer, 5 inner) + LOO	Groups matched for age, sex, sleep stage	SHAP: consistency between ML features and beta-band statistics	Available upon request
Mayor Torres et al. (2023) [50]	ADOS-2; 40 ASD, 48 TD adolescents	ASR; ADJUST; ZCA whitening	Leave-one-trial-out CV	ADOS-CS and IQ (KBIT-2) bias analysis	ROAR (LRP, SmoothGrad): late-stage FER relevance (500–1500 ms)	GitHub
Sharghilavan et al. (2024) [51]	Clinical evaluation; 13 ASD, 13 TD (7–12 yrs)	1–40 Hz; notch 50 Hz; 2 s epochs	80% training/20% test split	Matched for age, sex, handedness	SHAP/Grad-CAM: frontal-central regions reduce TD classification	Methods reported
Carson IV et al. (2024) [52]	DSM-5, ADI-R; 224 ASD, 69 NT (3–8 yrs)	HAPPE v3.2; CleanLine; average reference	Robustness R: 10× repeated 2-fold CV	Propensity-weighted sampling for age	Linear coefficients + SIRT: occipital gamma power/connectivity	Collaborative access
Dickinson et al. (2020) [32]	ADOS-T at 18 months; 65 high-risk infants	1–90 Hz; ASR; ICA; current source density	Nested LOOCV; permutation testing (1000)	MSEL-based control of cognitive bias	Forward modeling (Haufe): right TPJ connectivity predicts symptoms	Methods described
Shi et al. (2025) [53]	KAU (adolescents) + Sheffield (adults) datasets	0.5–100 Hz; ICA; notch 50/60 Hz; z-score	5-fold CV	Manual balancing of KAU ASD cases	SHAP: age-dependent shift (occipital vs. prefrontal)	Public datasets

**Table 3:** General characteristics of the studies. * Infant sample in [46] included high-risk ASD, high-risk language impairment, and low-risk infants. ** Data in [48] were aggregated from three independent datasets. *** Sample in [53] combined the Sheffield (28 adults with ASD and 28 healthy controls, aged 18–68 years) and KAU (12 children and adolescents with ASD, aged 6–20 years, and 5 TD controls aged 9–13 years) datasets.

Study	Country	Study Design	Sample	Age	EEG Condition	No. of Channels	Model Type	Main Outcome
Borra et al. (2022) [44]	Italy/Portugal	Computational, clinical feasibility	15 ASD	Mean: 22 years	Task-based (P300 BCI, VR)	8	DL (ICNN) + XAI	Right-parietal asymmetry of the P300 response; ICNN-derived features significantly correlated with ADOS-2 scores (r = 0.801).
Saranya & Menaka (2025) [45]	India	Case–control	10 ASD/10 TD	5–7 years	Task-based (viewing favorite cartoons)	3	ML (SVM) + XAI (SHAP)	The 12–16 Hz band yielded optimal discrimination; 93.59% classification accuracy using fuzzy entropy and DFA.
Irram & Suaib (2025) [46]	India	Longitudinal, observational	281 infants *	6 and 12 months	Resting-state (viewing of blown bubbles)	60–128	ML (SVM, RF, DT) + XAI (SHAP)	SVM achieved 93% AUC; elevated delta and gamma power in left frontal regions were the strongest predictors of ASD risk.
Wadhera & Mahmud (2023) [47]	India/UK	Case–control, computational	47 ASD/49 TD **	5–21 years	Resting-state (closed-eyes rest)	21	ML (SVM, KNN) + XAI (ELI5)	Reduced hierarchical network complexity in ASD (WHC: 0.55 vs. 0.78 in TD); 98.76% classification accuracy.
Wadhera (2023) [48]	India/UK	Case–control, computational	47 ASD/49 TD **	5–21 years	Resting-state (spontaneous EEG)	21	DL (Visible-GCN) + XAI	Visible-GCN achieved 93.78% accuracy; revealed hierarchical imbalances between sensory and associative brain networks in ASD.
Rogala et al. (2023) [49]	Poland/Belgium	Retrospective case–control	18 ASD/18 TD	2–6 years	Sleep (natural)	18	ML (Logistic Regression) + XAI (SHAP)	Accuracy 83–86%; PLV showed global hyperconnectivity, whereas ciPLV revealed hypoconnectivity in the low beta band.
Mayor Torres et al. (2023) [50]	Italy/USA	Case–control	40 ASD/48 TD	Mean: 15.34 years	Task-based (facial emotion recognition)	30	DL (CNN) + XAI (ROAR, LRP)	Group differences emerged mainly during late processing stages (500–1500 ms); ASD required additional temporal processing stages.
Sharghilavan et al. (2024) [51]	Iran/Romania/ Portugal/Sweden	Case–control	13 ASD/13 TD	7–12 years	Resting- and task-based (closed-eyes wakefulness and auditory perception task)	19	DL (Hybrid CNN–ResNet–BiLSTM) + XAI	Achieved 96.27% accuracy; SHAP highlighted frontal and central regions as most discriminative.
Carson IV et al. (2024) [52]	USA	Case–control (multi-study)	224 ASD/69 TD	3–8 years	Resting-state (viewing floating bubbles)	19	ML (Regularized GLM) + SIRT	Identified reproducible increases in gamma power and connectivity over occipital and posterior midline regions in ASD.
Dickinson et al. (2020) [32]	USA	Prospective longitudinal	65 infants	3 months (EEG); 18 months (ADOS-2)	Resting state (Spontaneous EEG viewing floating bubbles)	25	ML (SVR)	Alpha-band coherence at 3 months predicted ASD symptom severity at 18 months (r = 0.76).
Shi et al. (2025) [53]	China	Case–control, computational	40 ASD/33 TD ***	6–68 years	Resting-state closed-eyes	124–128	DL (TFSNet) + XAI (SHAP)	High classification accuracy (97–98%); SHAP revealed age-dependent spatial patterns (occipital in adults, prefrontal in adolescents).

**Table 4:** Sample clinical characteristics. * N/R: Not reported in the original article. ^1^ Sex distribution refers to the ASD or at-risk group when disaggregated data were available. ^2^ Diagnostic status distinguishes between clinically diagnosed ASD and infants classified as at risk based on familial or neurodevelopmental criteria. ^3^ Cognitive level is reported as Intelligence Quotient (IQ) or Developmental Quotient (DQ) assessed using standardized instruments (e.g., WAIS-III, Leiter-3, KBIT-2, MSEL). ^4^ Clinical tools include gold-standard diagnostic instruments such as ADOS-2, ADI-R, and MSEL. ^5^ Comorbidities and medications were frequently excluded or controlled for in computational EEG studies to reduce confounding effects on electrophysiological signals.

Study	Age Group	Sex (% Male in ASD/At-Risk) ^1^	Diagnostic Status ^2^	Cognitive Level (IQ/DQ) ^3^	ADOS-2/Clinical Tools ^4^	Comorbidities ^5^	Medications ^5^
Borra et al. (2022) [44]	Adults	N/R *	ASD (high-functioning)	FSIQ: 102.53 ± 11.24	ADOS-2 (Modules A, B, A+B), ADI-R, WAIS-III	N/R	N/R
Saranya & Menaka (2025) [45]	Children	N/R	ASD vs. TD	N/R	DSM-5, ISAA (Indian Scale for Assessment of Autism)	N/R	N/R
Irram & Suaib (2025) [46]	Infants	N/R	At-risk (HL-ASD) vs. TD (LL)	N/R	Language Development Survey (age 3)	N/R	N/R
Wadhera & Mahmud (2023) [47]	Children to adults	∼64% male (ASD)	ASD vs. TD	ASD FSIQ: 104.9 ± 7.3 (Dataset III)	ADOS-2, DSM-5, ICD-10	N/R	N/R
Wadhera (2023) [48]	Children to adults	∼75% male (ASD; Datasets I–II)	ASD vs. TD	N/R	ADOS-2	N/R	N/R
Rogala et al. (2023) [49]	Children	66.7% male	ASD vs. TD	12/18 within typical range (Leiter-3)	DSM-5, ICD-10, Leiter-3	Developmental regression (*n* = 4)	N/R
Mayor Torres et al. (2023) [50]	Adolescents	80% male	ASD vs. TD	High-functioning (KBIT-2)	ADOS-2, KBIT-2	N/R	N/R
Sharghilavan et al. (2024) [51]	Children	Matched with TD	ASD (high-functioning)	N/R	Clinical psychologist assessment	Excluded if present	Excluded if psychotropic
Carson IV et al. (2024) [52]	Children	∼75% male	ASD vs. TD	ASD DQ: 75.4 (Study 3); 92.3 (Study 4)	ADOS-2, ADI-R, DSM-5, MSEL	Epilepsy and Fragile X excluded	Stimulants (24 h washout in Study 1)
Dickinson et al. (2020) [32]	Infants	85.7% male (ASD1 group)	At-risk (familial risk)	Verbal T-score: 31.75 (MSEL)	ADOS-T (Toddler Module), MSEL	N/R	N/R
Shi et al. (2025) [53]	Adolescents/adults	N/R	ASD vs. healthy controls	N/R	Professional clinical scales	N/R	N/R

**Table 5:** Explainable AI methods and interpretability outcomes across the included studies.

Study	Model	XAI Method	Level of Interpretability	Clinically Interpretable Output	Clinical Validation
Borra et al. (2022) [44]	ICNN (Sinc-ShallowNet-v2)	Saliency maps/gradients	Spatial/Spectral	Right-parietal asymmetry (P4) in P300 responses during social attention tasks.	Correlation with ADOS-2 scores (r = 0.801).
Saranya & Menaka (2025) [45]	SVM	SHAP	Spectral/Feature-based	12–16 Hz band as most discriminative for ASD classification.	Consistency with ISAA scores and DSM-5 criteria.
Irram & Suaib (2025) [46]	SVM/RF/DT	SHAP	Spatial/Spectral	Elevated delta and gamma power in left frontal regions as early predictors of ASD risk.	Longitudinal validation (Language Development Survey at 3 years).
Wadhera & Mahmud (2023) [47]	SVM/KNN	ELI5	Network-level (structural)	Reduced WHC in ASD networks; fewer hierarchical levels than TD networks.	Diagnostic standardization based on ADOS-2.
Wadhera (2023) [48]	VGCN	Integrated Gradients	Spatial (nodes/edges)	Hierarchical imbalances between sensory and associative networks.	Benchmark vs. other models; no direct clinical-score correlation.
Rogala et al. (2023) [49]	Logistic Regression	SHAP	Spatial/Spectral	PLV hyperconnectivity and ciPLV hypoconnectivity in low beta band.	DSM-5/ICD-10 clinical diagnosis.
Mayor Torres et al. (2023) [50]	CNN	LRP-B, PatternNet, SmoothGrad	Temporal/Spatial	Additional late stages (500–1500 ms) for negative facial emotions in ASD.	Severity assessed with ADOS-2.
Sharghilavan et al. (2024) [51]	Hybrid CNN–ResNet–BiLSTM	SHAP, Grad-CAM	Spatial/Spectral	Frontal/central regions strongest negative contribution to “typical” class.	Clinical evaluation by certified psychologists.
Carson IV et al. (2024) [52]	Regularized GLM	SIRT	Spatial/Spectral	Reproducible increased occipital gamma activity associated with ASD.	Consistency across trajectories and age analyses.
Dickinson et al. (2020) [32]	SVR	Haufe patterns	Spatial/Spectral	Reduced frontal and increased right TPJ connectivity at 3 months.	Prediction of ADOS-T at 18 months (r = 0.76).
Shi et al. (2025) [53]	TFSNet	SHAP	Spatial/Temporal/Spectral	Occipital dominance in adults; prefrontal dominance in adolescents.	Aligned with reported developmental differences.

**Table 6:** Summary of electrophysiological findings distinguishing ASD and TD. * ↑ ASD/↓ ASD: Increased or decreased feature in ASD relative to TD individuals.

Study	Domain	EEG Feature	Direction of Effect (ASD vs. TD) *	Regions/Frequency Bands	Age Group	Functional Interpretation
Borra et al., 2022 [44]	Task-based (P300)	Spatial relevance (ICNN)	Asymmetry	Right parietal region (P4)	Adults (mean: 22 yrs)	Atypical visuospatial and social stimulus processing, reflected in altered parietal engagement during P300 responses.
Irram & Suaib, 2025 [46]	Spectral	Power spectral density (PSD)	↑ ASD	Left frontal/Delta and Gamma	Infants (6–12 months)	Early electrophysiological markers predictive of ASD risk prior to overt behavioral symptoms.
Saranya & Menaka, 2025 [45]	Spectral	Frequency band discrimination	Optimal band	12–16 Hz (high alpha/low beta)	Children (5–7 yrs)	Identification of the most discriminative oscillatory range for objective ASD–TD classification.
Wadhera & Mahmud, 2023 [47]	Connectivity/Network	Weighted Hierarchical Complexity (WHC)	↓ ASD	Whole network (up to 3 hierarchical levels)	Children to adults (5–21 yrs)	Attenuated network hierarchy with reduced global integration and predominance of local connectivity.
Wadhera, 2023 [48]	Network (VGCN)	Hierarchical imbalance	Pronounced	Visibility-graph-based network topology	Children to adults (5–21 yrs)	Disrupted hierarchical organization between sensory and associative networks, potentially impairing cognitive segregation.
Rogala et al., 2023 [49]	Connectivity	Phase coherence (PLV vs. ciPLV)	↑ ASD (PLV), ↓ ASD (ciPLV)	Frontal/centro-parietal; low beta	Children (2–6 yrs)	Apparent hyperconnectivity driven by zero-lag coupling, alongside reduced genuine non-zero-lag interactions.
Mayor Torres et al., 2023 [50]	Task-based (FER)	Temporal relevance dynamics	↑ ASD (additional stages)	Late window 500–1500 ms	Adolescents (mean: 15.3 yrs)	Altered and prolonged temporal dynamics during emotional processing, particularly for negative emotions.
Sharghilavan et al., 2024 [51]	Spectral/Task-based	Regional relevance (SHAP)	↑ ASD	Central and frontal regions	Children (7–12 yrs)	Reduced efficiency in complex information integration and reliance on additional processing stages during speech tasks.
Carson IV et al., 2024 [52]	Spectral	Gamma-band power	↑ ASD	Occipital and posterior midline regions	Children (3–8 yrs)	Reproducible marker of altered excitation/inhibition balance, particularly in visual processing networks.
Dickinson et al., 2020 [32]	Connectivity	Alpha-band phase coherence (6–12 Hz)	↓ Frontal/↑ Right TPJ	Frontal cortex vs. temporoparietal junction	Infants (3 months)	Early predictor of later ASD symptom severity; increased TPJ coherence may reflect network inefficiency or hub overload.
Shi et al., 2025 [53]	Spectral/Spatial	Regional contribution (SHAP)	Age-dependent	Occipital (adults)/Prefrontal (adolescents)	6–68 yrs	Developmental shift from altered visual integration to executive-function-related deficits.

## Data Availability

No new data were created in this study.

## Supplementary Materials

The following supporting information can be downloaded at: https://www.mdpi.com/article/10.3390/neurosci7020041/s1, Table S1: Preferred Reporting Items for Systematic Reviews and Meta-Analyses (PRISMA) 2020 checklist; Table S2: Preferred Reporting Items for Systematic Reviews and Meta-Analyses (PRISMA) 2020 abstract checklist.

## Abbreviations

The following abbreviations are used in this manuscript:

ADHD	Attention-Deficit/Hyperactivity Disorder
ADI-R	Autism Diagnostic Interview—Revised
ADOS-2	Autism Diagnostic Observation Schedule
ADOS-T	Toddler Module of ADOS-2
AI	Artificial Intelligence
ASD	Autism Spectrum Disorder
AUC	Area Under the Curve
BCI	Brain–Computer Interface
ciPLV	Corrected Imaginary Phase Locking Value
CNN	Convolutional Neural Network
DFA	Detrended Fluctuation Analysis
DL	Deep Learning
DQ	Developmental Quotient
DSM-5	Diagnostic and Statistical Manual of Mental Disorders—Fifth Edition
DT	Decision Tree
EEG	Electroencephalography
ELI5	Explain Like I’m 5
ERP	Event-Related Potential
FER	Facial Emotion Recognition
GLM	Generalized Linear Model
Grad-CAM	Gradient-weighted Class Activation Mapping
HL	High Likelihood
Hybrid CNN-ResNet-BiLSTM	Convolutional Neural Network—Residual Network—Bidirectional
	Long Short-Term Memory
ICD-10	International Classification of Diseases, 10th Revision
ICNN	Interpretable Convolutional Neural Network
IQ	Intelligence Quotient
ISAA	Indian Scale for Assessment of Autism
KBIT-2	Kaufman Brief Intelligence Test—Second Edition
k-NN	k-Nearest Neighbors
Leiter-3	Leiter International Performance Scale—Third Edition
LL	Low Likelihood
LRP	Layer-wise Relevance Propagation
LRP-B	Layer-wise Relevance Propagation—Beta rule
LSTM	Long Short-Term Memory
ML	Machine Learning
MSEL	Mullen Scales of Early Learning
NN	Neural Network
PLV	Phase Locking Value
PSD	Power Spectral Density
RF	Random Forest
ROAR	Remove And Retrain
SHAP	SHapley Additive exPlanations
SIRT	Simultaneous Iterative Reconstruction Technique
SVM	Support Vector Machine
TD	Typically Developing
TFSNet	Time–Frequency Synergy Network
TPJ	Temporal Parietal Junction
VGCN	Visible Graph Convolutional Network
VR	Virtual Reality
WAIS-III	Wechsler Adult Intelligence Scale—Third Edition
WHC	Weighted Hierarchical Complexity
XAI	Explainable Artificial Intelligence
